# Development and validation of a nomogram to predict failure of 14-day negative nucleic acid conversion in adults with non-severe COVID-19 during the Omicron surge: a retrospective multicenter study

**DOI:** 10.1186/s40249-023-01057-4

**Published:** 2023-02-07

**Authors:** Honglian Gui, Zhenglan Zhang, Bin Chen, Yaoxing Chen, Yue Wang, Zhuo Long, Chuanwu Zhu, Yinling Wang, Zhujun Cao, Qing Xie

**Affiliations:** 1 Department of Infectious Diseases, Ruijin Hospital, Shanghai Jiao Tong University School of Medicine, Shanghai, China; 2 Department of Infectious Diseases, Shanghai Pudong Hospital, Fudan University Pudong Medical Center, Shanghai, China; 3 Department of Hepatology, Wuwei People’s Hospital, Gansu, China; 4 Department of Infectious Diseases, The Affiliated Infectious Diseases Hospital of Soochow University, Suzhou, China; 5 Department of Endocrine and Metabolic Diseases, Fengcheng Hospital of Fengxian District, Shanghai, China

**Keywords:** Nomogram, Viral shedding time, Prediction, Omicron variant, COVID-19

## Abstract

**Background:**

With the variability in emerging data, guidance on the isolation duration for patients with coronavirus disease 2019 (COVID-19) due to the Omicron variant is controversial. This study aimed to determine the predictors of prolonged viral RNA shedding in patients with non-severe COVID-19 and construct a nomogram to predict patients at risk of 14-day PCR conversion failure.

**Methods:**

Adult patients with non-severe COVID-19 were enrolled from three hospitals of eastern China in Spring 2022. Viral shedding time (VST) was defined as either the day of the first positive test or the day of symptom onset, whichever was earlier, to the date of the first of two consecutively negative PCR tests. Patients from one hospital (Cohort I, *n* = 2033) were randomly grouped into training and internal validation sets. Predictors of 14-day PCR conversion failure were identified and a nomogram was developed by multivariable logistic regression using the training dataset. Two hospitals (Cohort II, *n* = 1596) were used as an external validation set to measure the performance of this nomogram.

**Results:**

Of the 2033 patients from Cohort I, the median VST was 13.0 (interquartile range: 10.0‒16.0) days; 716 (35.2%) lasted > 14 days. In the training set, increased age [per 10 years, odds ratio (*OR*) = 1.29, 95% confidence interval (*CI*): 1.15‒1.45, *P* < 0.001] and high Charlson comorbidity index (*OR* = 1.25, 95% *CI*: 1.08‒1.46, *P* = 0.004) were independent risk factors for VST > 14 days, whereas full or boosted vaccination (*OR* = 0.63, 95% *CI*: 0.42‒0.95, *P* = 0.028) and antiviral therapy (*OR* = 0.56, 95% *CI*: 0.31‒0.96, *P* = 0.040) were protective factors. These predictors were used to develop a nomogram to predict VST > 14 days, with an area under the ROC curve (AUC) of 0.73 in the training set (AUC, 0.74 in internal validation set; 0.76 in external validation set).

**Conclusions:**

Older age, increasing comorbidities, incomplete vaccinations, and lack of antiviral therapy are risk factors for persistent infection with Omicron variant for > 14 days. A nomogram based on these predictors could be used as a prediction tool to guide treatment and isolation strategies.

**Graphical Abstract:**

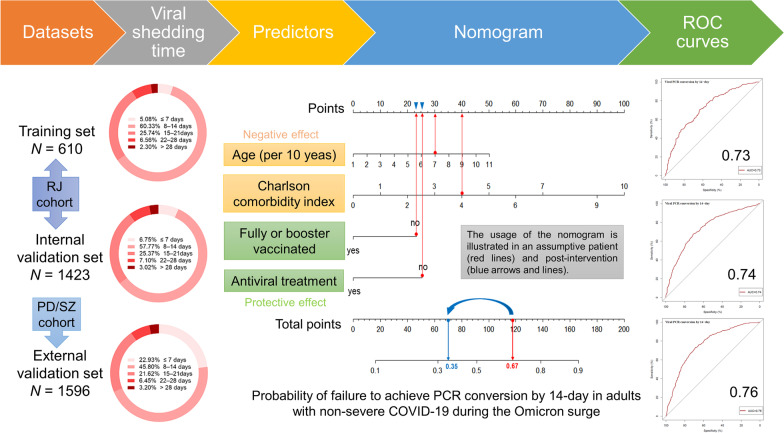

## Background

Currently, the Omicron variant of severe acute respiratory syndrome coronavirus 2 (SARS-CoV-2) has rapidly and exponentially spread globally since its first identification in specimens collected in November 2021 [1]. Affected by the global spread of Omicron, an outbreak of coronavirus disease 2019 (COVID-19) caused by the Omicron BA.2.2 subvariant emerged in Shanghai, China during Spring 2022, which involved 593336 cases by May 4 [2]. Although Omicron generally causes less clinically severe illnesses, it remains a formidable challenge for public health due to its high transmissibility [3, 4].

Given the potentially profound impact of transmission-based precautions on patients, their families, hospital systems, and public health, extending or shortening their duration can be difficult [5]. Since the end of 2021, the US Centers for Disease Control and Prevention recommended shortening the isolation period for infected individuals from 10 to 5 days after symptom onset or after testing positive [6]. The World Health Organization updated its recommendation on April 12, 2022, stating that symptomatic individuals should remain isolated for at least 10 days after symptom onset, plus another three days following symptoms end, whereas asymptomatic individuals should stay isolated for at least 10 days after testing positive [7]. Policies on the duration of inpatient and outpatient isolation for people with the Omicron variant infections have been controversial given the variability in emerging data. Therefore, there continues to be uncertainty regarding the clinical significance of prolonged PCR positivity and various routes of viral shedding. It is critical to understand the duration and predictors of viral shedding in patients with non-severe COVID-19 for optimizing treatment options and preventing the transmission of this disease.

In this study, we performed a retrospective multicenter study to investigate trends in clinical illness and PCR-based viral shedding duration and to identify risk factors influencing persistent RNA shedding in adults with non-severe COVID-19 during the Omicron surge. We further constructed a predictive nomogram model to identify patients at risk of persistent infection exceeding 14 days. Such a model may be beneficial for decision-making regarding epidemic prevention and control.

## Methods

### Study design and population

The study protocol was approved by the Research Ethics Committee of Ruijin Hospital (No. 2022-LLS-63). Patient informed consents were exempted on account of the retrospective nature of this study.

This retrospective study was performed in three COVID-19 designated hospitals in eastern China: Shanghai Ruijin Hospital, Shanghai Pudong Hospital, and Suzhou Fifth People’s Hospital. Patients diagnosed with SARS-CoV-2 infection upon hospital admission during the indicated periods were consecutively screened for study eligibility for this study. Cohort I was obtained from Shanghai Ruijin Hospital between March 17, 2022 and May 16, 2022. Cohort II was obtained from Shanghai Pudong Hospital (between March 23, 2022 and May 31, 2022) and Suzhou Fifth People’s Hospital (between February 10, 2022 and May 31, 2022). The exclusion criteria for participants were as follows: (1) age < 18 years on admission; (2) the negative viral PCR on the day of admission; (3) loss to follow-up or transfer to other designated hospitals; (4) incomplete vaccine information in their medical records; (5) absence of chest computed tomography (CT) scan results to assess disease severity; and (7) diagnosis of severe or critical COVID-19 during hospitalization. Patients with severe/critical COVID-19 were excluded, as lower respiratory tract specimens were collected for patients with mechanical ventilation; a bias might be introduced when comparing direct viral RNA shedding between the upper and lower respiratory tract specimens [8].

### Data collection and study outcomes

All data (demographic, clinical, biochemical, virological, and radiological features) were extracted retrospectively from the hospital electronic patient database, including age, gender, body mass index, vaccination status, comorbidities, symptoms, laboratory results, chest CT scan results, and antiviral medications (nirmatrelvir/ritonavir or VV116, if available).

Day 0 was defined as the day of the first positive test or the day of symptom onset, whichever occurred earlier. The SARS-CoV-2 viral load was detected and quantified using nasopharyngeal and oropharyngeal swabs on alternate days after hospital admission. The cycle threshold (Ct) value from qRT-PCR was used to represent the viral load of SARS-COV-2. A Ct value of > 35 for both ORF1ab and N genes was considered negative in this study. Negative nucleic acid test conversion was defined as two consecutive negative PCR results > 24 h apart, thereby meeting the criteria for deisolation. Viral shedding time (VST) was defined as the time from day 0 to the first date of these two consecutive negative tests. The primary outcome was a VST longer than 14 days.

### Diagnosis and clinical evaluation

The vaccination status of the individuals varied according to different vaccine types and was divided into four categories: unvaccinated, partially vaccinated, fully vaccinated, and booster vaccinated. Briefly, all three conditions were defined as full vaccination: one single dose of adenoviral vectored vaccine, two doses of inactivated or mRNA vaccine, and three doses of protein-subunit vaccine.

We collected data regarding underlying medical conditions, including cardiocerebrovascular disease, diabetes mellitus, chronic lung disease, chronic hepatic disease, chronic kidney disease, and malignancy. We also collected information regarding the presence of an immunocompromised state (*e.g.*, human immunodeficiency virus infection, chronic use of corticosteroids, or use of other immunosuppressive drugs). Charlson comorbidity index (CCI) scores were calculated by weighting and scoring comorbid conditions, as previously reported by Charlson [9]. As age may be an independent factor, an age-adjusted CCI score was not used in the present study [10].

The clinical severity of these cases was classified as asymptomatic, mild, moderate, severe, and critical COVID-19, according to the 9th version of the national COVID-19 protocol [11]. Asymptomatic, mild, and moderate infections were considered non-severe infections. Symptomatic infections were defined as those with COVID-19–related symptoms at any point during the observation period.

### Statistical analysis

Statistical analyses were performed using the SPSS software (version 23 for Windows; IBM Corp., Armonk, NY, USA) and R software (version V.3.6.2, http://cran.r-project.org). Two-sided *P* values < 0.05 were statistically significant. Continuous variables were presented as median and interquartile range (IQR) and were compared using the Mann–Whitney *U* test. Frequency variables were expressed as numbers and percentages and compared using the chi-squared test. After enrollment, patients from Cohort I were randomly grouped at a ratio of 3:7 into the training and internal validation sets, whereas patients from Cohort II were used as the external validation set. The VST distribution was visualized using boxplots and pie plots according to the different datasets. The significance of demographic and clinical variables was assessed using univariate logistic models, followed by multivariate adjustment to identify independent risk factors for VST > 14 days in the training set. Odds ratio (*OR*) was reported with 95% confidence interval (*CI*). These variables were also tested in a time-to-event analysis using the Kaplan–Meier plot with the log-rank test as a sensitivity analysis. All variables at a statistically significant level (*P* < 0.05) after multivariate analysis were used to establish a nomogram. The receiver operating characteristic (ROC) curve and the area under the ROC curve (AUC) were used to assess the discrimination of the model, while the calibration plot was used to graphically evaluate the calibration of the nomogram. Calibration plots showed the apparent (actual), bias-corrected (adjusted), and ideal (100% agreement) curves with bootstrapping. The discrimination and calibration of the nomogram were validated using the internal and external validation sets.

## Results

### Clinical characteristics of patients

**Fig. 1:**
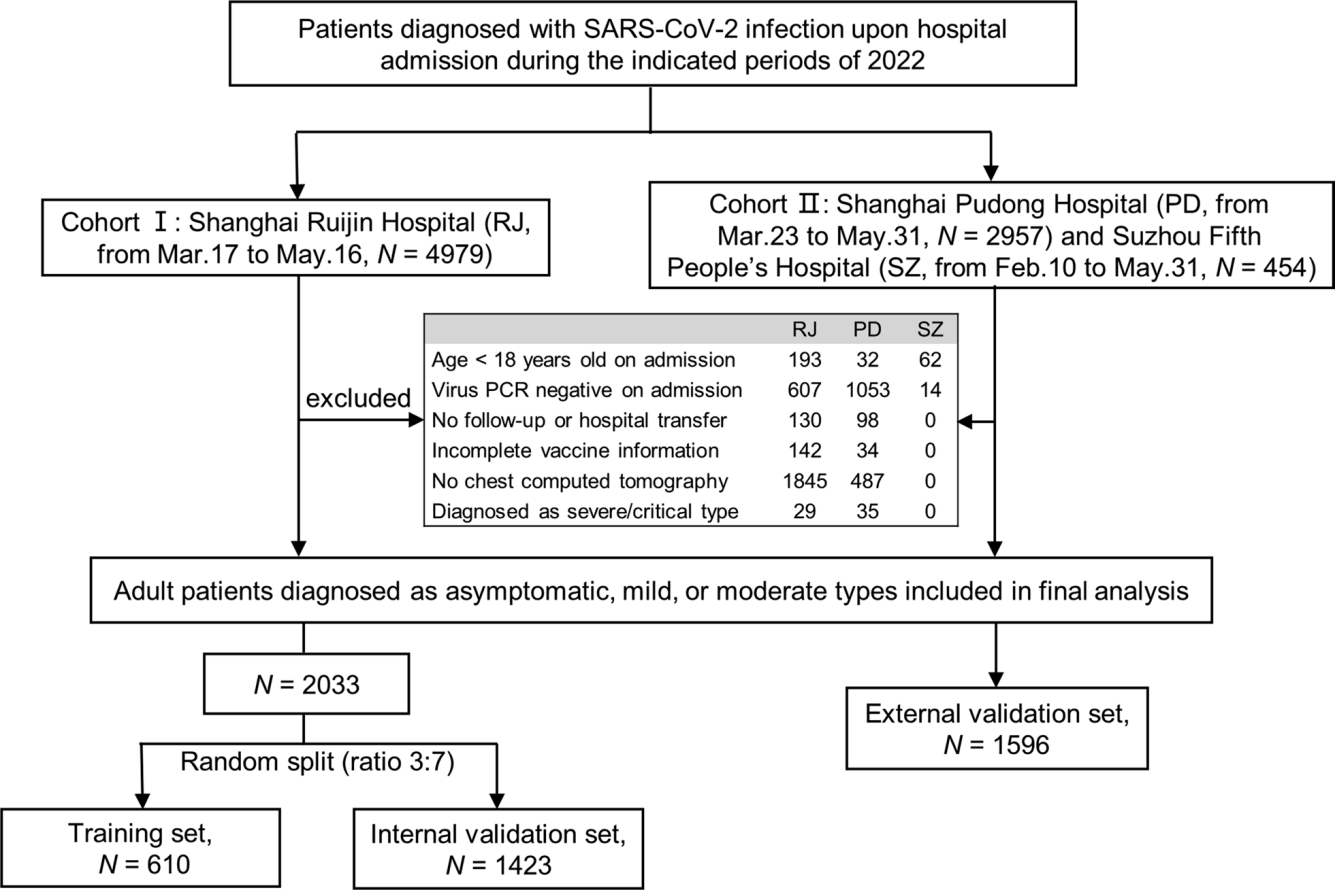
Flow chart of study participants

A total of 8390 patients were identified and diagnosed with SARS-CoV-2 infection during the indicated study periods; 4761 patients were sequentially excluded according to the exclusion criteria, the majority due to the negative virus PCR on admission, and absence of chest CT scan during admission (Fig. 1). Finally, 3629 adults diagnosed with asymptomatic, mild, or moderate types were included and analyzed. Cohort I was randomly split in a 3:7 ratio into the training (*n* = 610) and internal validation (*n* = 1423) sets. Cohort II was used as the external validation set (*n* = 1596).

Among the 2033 patients from Cohort I, 1040 (51.2%) were male, with a median age of 59.0 (IQR: 43.0–70.0) years, with 994 (48.9%) aged ≥ 60 years. A total of 1107 (54.5%) patients had received a full or booster vaccination for COVID-19, whereas 859 (42.3%) had not been vaccinated. According to the symptoms and imaging findings, 586 (28.8%) were asymptomatic, 890 (43.8%) were mild, and 557 (27.4%) were moderate. The median CCI score was 0 (IQR: 0–2), and 1130 (55.6%) patients had one or more underlying comorbidities: hypertension (691, 34.0%) was the most common, followed by other cardio-cerebrovascular diseases (374, 18.4%), and diabetes (296, 14.6%). In total, 12.1% (247/2033) of patients were treated with either nirmatrelvir/ritonavir or VV116 during admission. The clinical characteristics of patients from the different analysis sets are presented in Table 1. None of the clinical characteristics were significantly different between the training and internal validation sets (all *P* > 0.05).

**Table 1:** Demographic and clinical features of patients in the training and validation sets

	Training set	Internal validation set	External validation set
Number of patients, *n*	610	1423	1596
Age, years	59.5 (42.0–70.0)	59.0 (44.0–71.0)	60.0 (43.0–74.0)
Male,* n* (%)	331 (54.3)	709 (49.8)	832 (52.1)
Body mass index, kg/m^2^	23.3 (21.2–26.0)	23.4 (21.2–25.6)	23.3 (20.9–25.7)
Vaccination status, *n* (%)			
Unvaccinated	243 (39.8)	616 (43.3)	678 (42.5)
Partially vaccinated	25 (4.1)	42 (3.0)	50 (3.1)
Fully vaccinated	154 (25.2)	335 (23.5)	453 (28.4)
Boosted	188 (30.8)	430 (30.2)	415 (26.0)
Symptomatic, *n* (%)	367 (60.2)	878 (61.7)	932 (58.4)
Disease severity, *n* (%)			
Asymptomatic/mild	456 (74.8)	1020 (71.7)	1091 (68.4)
Moderate	154 (25.2)	403 (28.3)	505 (31.6)
Co-existing conditions, *n* (%)			
Hypertension	215 (35.2)	476 (33.5)	516 (32.3)
Cardio-cerebrovascular disease excluding hypertension	122 (20.0)	252 (17.7)	261 (16.4)
Diabetes	95 (15.6)	201 (14.1)	246 (15.4)
Chronic lung disease	43 (7.0)	110 (7.7)	88 (5.5)
Chronic liver disease	19 (3.1)	46 (3.2)	34 (2.1)
Chronic kidney disease	30 (4.9)	83 (5.8)	95 (6.0)
Malignancy	43 (7.0)	108 (7.6)	113 (7.1)
Immunol suppressed status	17 (2.8)	42 (3.0)	27 (1.7)
Charlson comorbidity index	0.0 (0.0–2.0)	0.0 (0.0–2.0)	0.0 (0.0–1.0)
Antiviral treatment, *n* (%)	78 (12.8)	169 (11.9)	154 (9.6)

Continuous variables are reported as the median and interquartile range

### Viral shedding time and its predictors

**Fig. 2:**
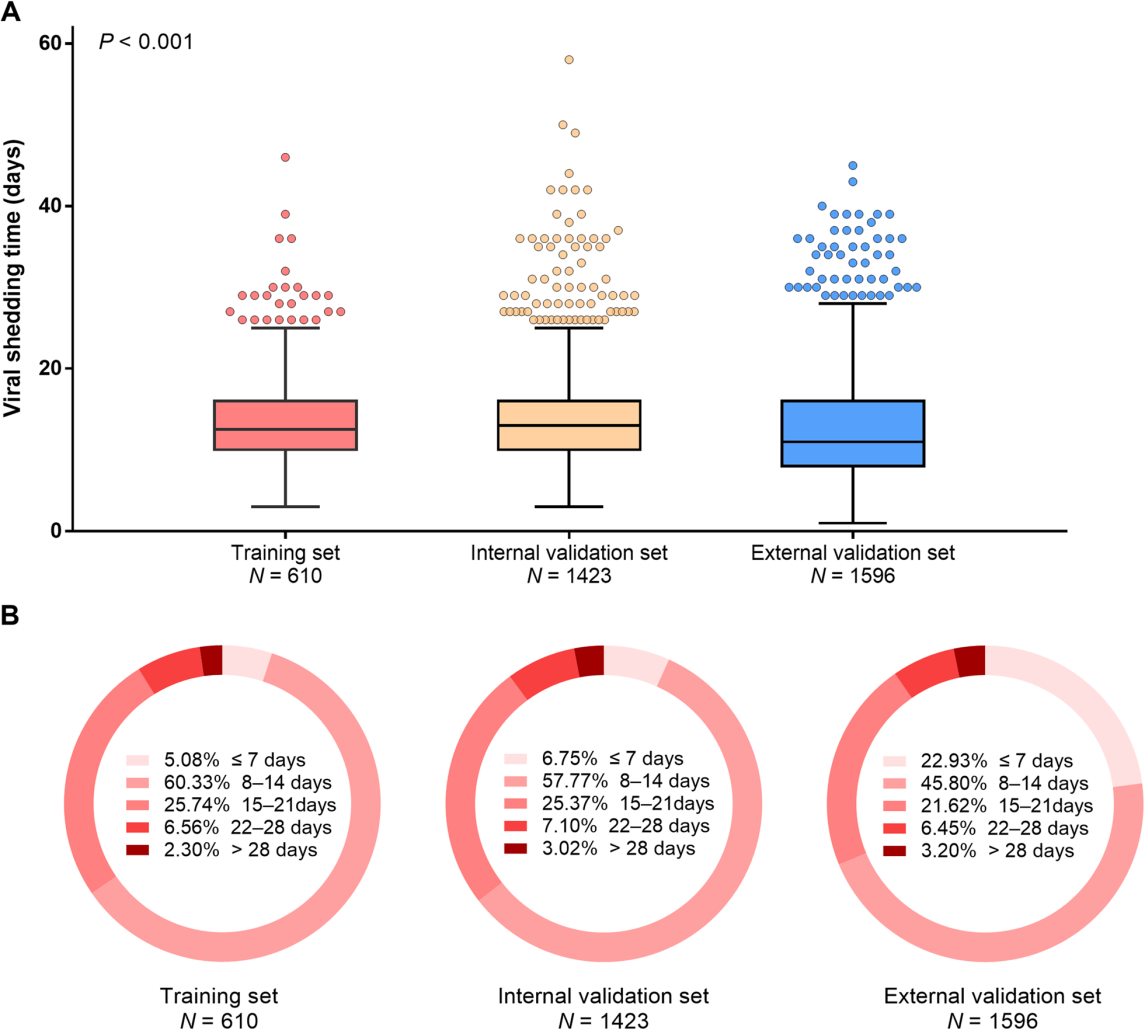
Viral shedding time according to different analysis sets. Boxplot with median, 25th and 75th percentiles (**A**); Percentages of viral conversion on PCR test by weeks (**B**)

Of all 2033 patients from Cohort I, the median VST was 13.0 (IQR: 10.0–16.0) days; 1317 (64.8%) occurred within 14 days, 659 (32.4%) between 14 and 28 days, and even 57 (2.8%) beyond 28 days. The boxplot with median VST and its distribution according to different analysis sets were shown in Fig. 2.

**Fig. 3:**
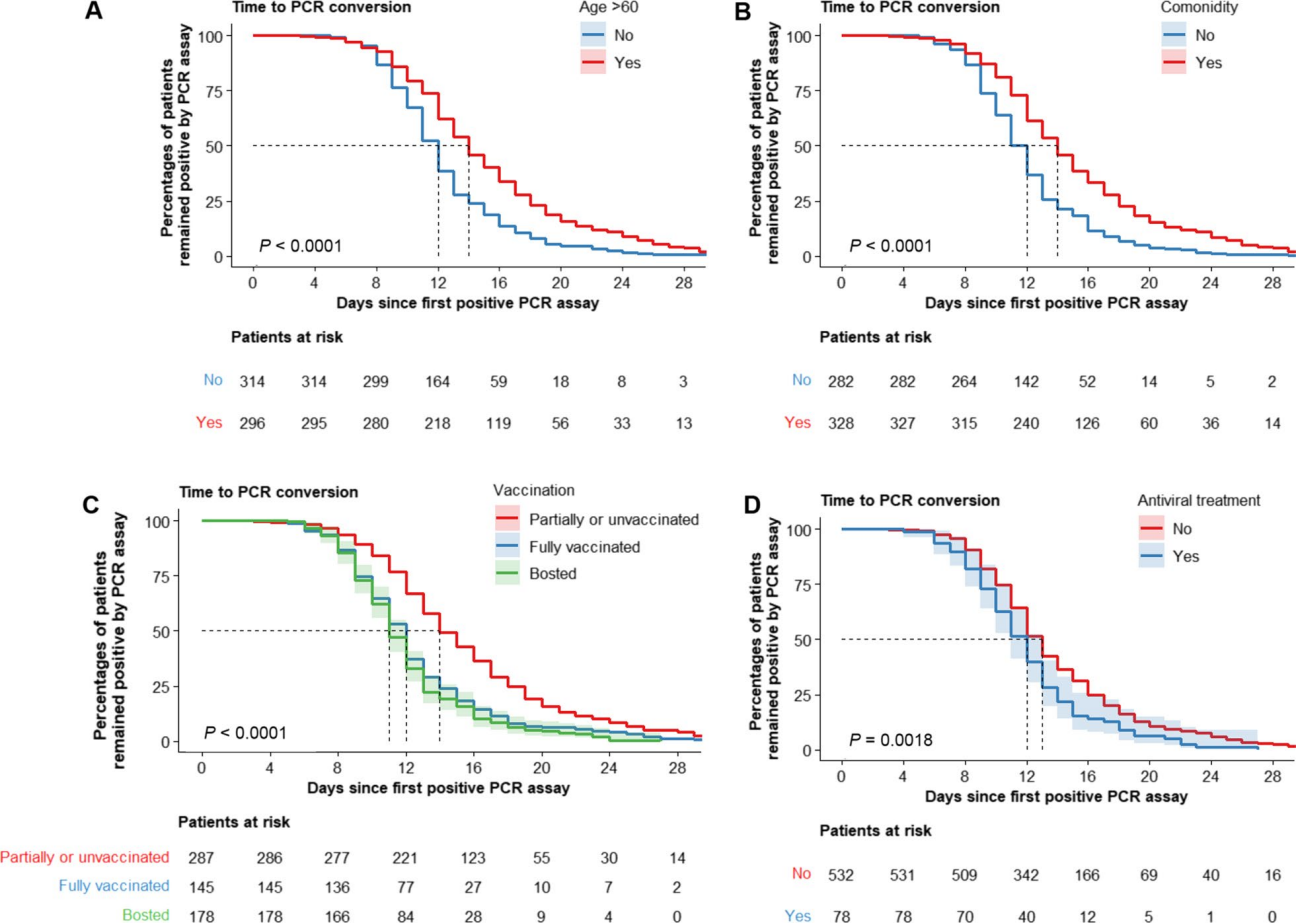
Kaplan–Meier survival curves for the viral shedding time. Kaplan–Meier survival curves demonstrate the viral shedding time in the training set, according to age (**A**), comorbidity (**B**), vaccination status (**C**), and antiviral treatment (**D**), respectively

As indicated by the Kaplan–Meier curves, viral RNA conversion times in the training set were reduced in individuals < 60 years, without comorbidities, with full or booster vaccination, and treated with antiviral medications (Fig. 3). In the training set, univariable and multivariable logistic regression showed that the four independent predictors for VST > 14 days were increased age, lack of full vaccination, high CCI score, and absence of antiviral treatment (Table 2).

**Table 2:** Univariate and multivariate logistic regression analyses for the risk factors associated with viral shedding time > 14 days in the training set

	Univariate		Multivariate	
	*OR* (95% *CI*)	*P* value	*OR* (95% *CI*)	*P* value
Age (per 10 years)	1.503 (1.358–1.671)	< 0.001	1.293 (1.147–1.463)	< 0.001
Gender				
Female	1.00	-		
Male	1.010 (0.724–1.411)	0.954		
Body mass index (per point)	0.986 (0.940–1.034)	0.564		
Vaccination				
Reference*	1.00	-	1.00	-
Fully vaccinated	0.270 (0.170–0.420)	< 0.001	0.629 (0.416–0.952)	0.028
Boosted	0.278 (0.182–0.420)	< 0.001		
Symptomatic				
No	1.00	-		
Yes	0.827 (0.590–1.161)	0.272		
Disease severity				
Reference^†^	1.00	-	1.00	-
Moderate	2.143 (1.475–3.116)	< 0.001	1.155 (0.749–1.772)	0.511
Charlson comorbidity index (per point)	1.581 (1.390–1.811)	< 0.001	1.247 (1.075–1.455)	0.004
Antiviral treatment				
No	1.00	-		
Yes	0.570 (0.331–0.949)	0.036	0.558 (0.314–0.961)	0.040

*OR* odds ratio, *CI* confidence interval

*Patients remained unvaccinated or only partially vaccinated were considered as reference group

^†^Patients with asymptomatic/mild type were considered as reference group

### Predictive nomogram for the probability of VST > 14 days

**Fig. 4:**
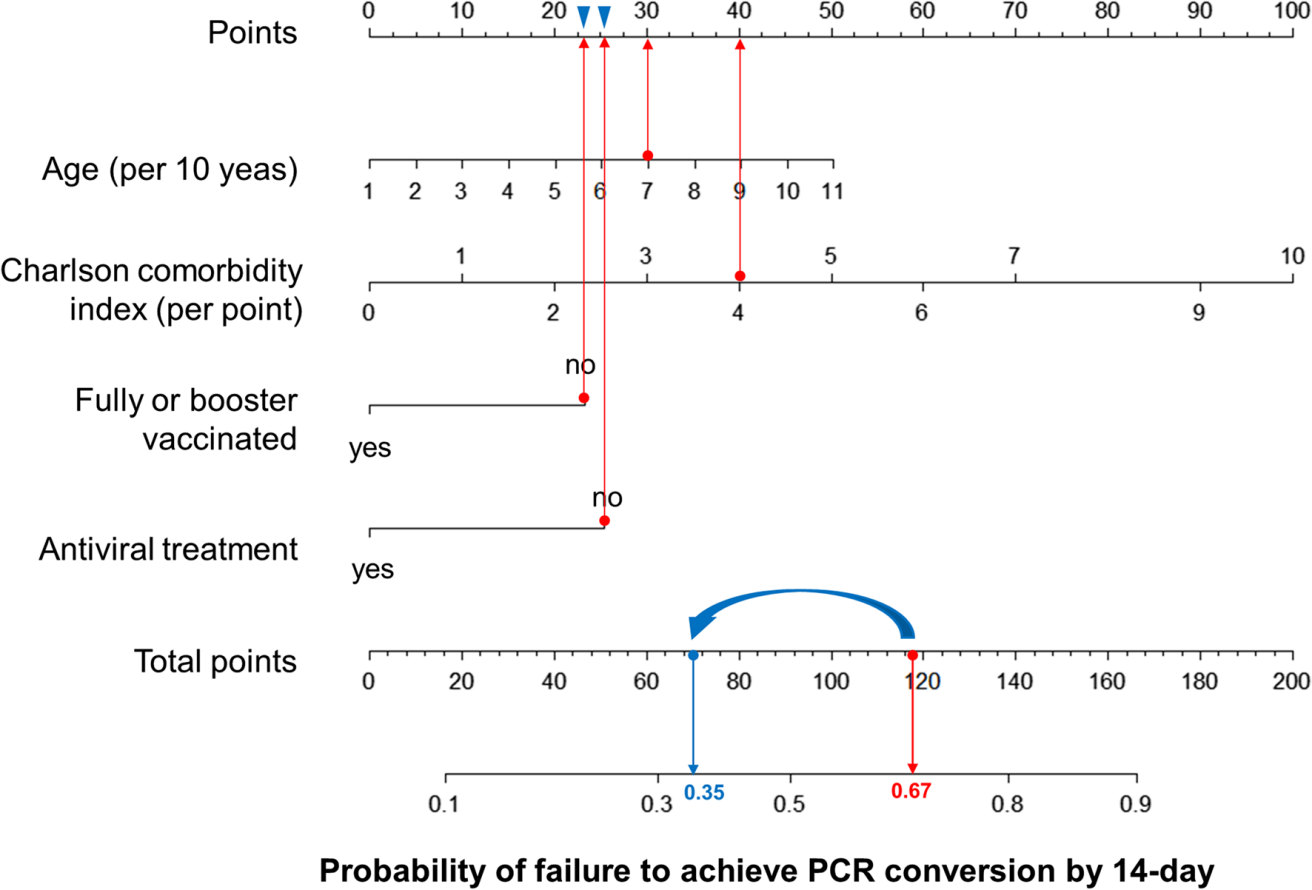
The prediction nomogram in adults with non-severe COVID-19. The nomogram composed of age, Charlson comorbidity index, fully vaccinated or booster, and antiviral treatment was developed to predict the probability of failure to achieve PCR conversion by 14-day. The usage of the nomogram is illustrated in an assumptive patient (red lines) and post-intervention (blue arrows and lines)

A predictive nomogram was formulated based on the independent predictors of VST > 14 days from the training cohort (Fig. 4). Its utility is illustrated by a 70-year-old assumptive patient with cirrhosis and diabetes, unvaccinated, and no antiviral therapy on admission (red lines). The total number of points was 118 for this patient, which represents the 0.67 probability of failure to achieve PCR conversion within 14 days.

**Fig. 5:**
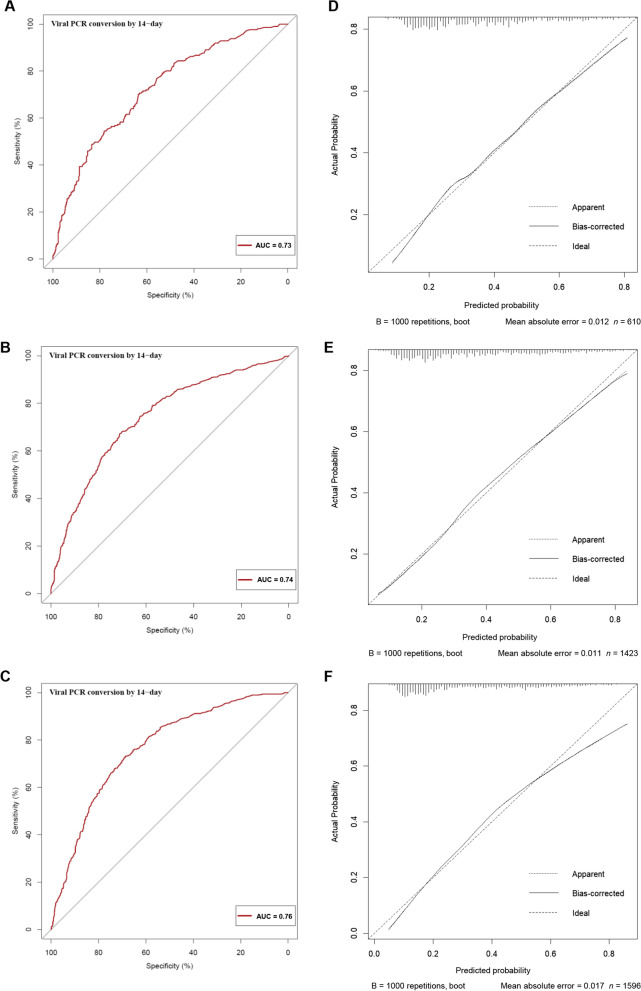
The calibration and ROC curves of the nomogram. The ROC and calibration curves for performance to distinguish individuals with VST > 14 days from with VST ≤ 14 days in the training set (**A**, **D**), validation set (**B**, **E**), and external validation set (**C**, **F**), respectively. *AUC* area under the ROC curve, *ROC* receiver operating characteristic, *VST* viral shedding time

The performance of this nomogram was assessed using calibration plots and the AUC (Fig. 5). The calibration plots showed adequate agreement between the predictive nomogram and actual observations in the training, internal, and external validation sets, indicating a robust calibration of the nomogram. Furthermore, AUC for predicting VST > 14 days was 0.73 in the training set, and was 0.74 and 0.76 in the internal and external validation sets, respectively, indicating an acceptable discrimination of the nomogram.

## Discussion

Data on the clinical course, particularly on the VST of non-severe SARS-CoV-2 infections, are of paramount importance to optimize treatment options and prevent transmission of the disease. In this study, we developed and validated a nomogram for predicting 14-day PCR conversation failure in patients with non-severe COVID-19 from the Omicron variant. The nomogram, which was based on age, CCI score, vaccine status, and antiviral usage, showed acceptable discrimination in predicting a VST > 14 days. Thus, access to vaccinations and antiviral therapies is crucial to protect older and vulnerable individuals and prevent overwhelming healthcare system.

More than half of the infections caused by the Omicron variant in our study remained viral PCR-positive after the recommended 5- or 10-day isolation period. One reason for this may be that the recommended duration of isolation was based on viral culture testing rather than PCR testing, in which the viral loads differed [5, 12]. Another reason may be that many asymptomatic or pre-symptomatic patients were identified by expanding RNA tests in accordance with the local dynamic zero-COVID policy [2]. Omicron presents a shorter incubation period of two to four days than the previous variants [13]. Collectively, the present study used a 14-day VST as a cut-off, and over one-third of the participants experienced VSTs > 14 days.

It is difficult to justify whether a symptom-based or test-based strategy is more suitable for determining the isolation duration of infected individuals. Prediction models may aid in identifying patients with prolonged SARS-CoV-2 RNA shedding from the respiratory tract. To make the prediction model simple and easy to use in the clinical setting, we focused on risk factors that did not require complex laboratory parameters. Four characteristics emerged as significant independent predictors of prolonged VST: old age and increasing comorbidities, which both showed a negative effect, and vaccines and antiviral therapies, which displayed positive effect.

In our study, the median VST was 13.0 days. This result differs from those of previous studies conducted during the Shanghai Omicron outbreak [14, 15]. Data from a Fangcang shelter showed an average length of hospital stay of 7.18 ± 3.05 days [14]. It should, however, be noted that the length of hospital stay is not synonymous with the VST. The VV116 trial (mean age: 33.9 years old) showed VSTs of 9.92 and 11.13 days in the VV116 treatment and control groups, respectively [15]. Patients recruited from designated hospitals tend to be older and have more underlying illnesses than those recruited from shelters and those participating in clinical trials. Older age and the presence of comorbidities are well-known risk factors associated with prolonged VST and COVID-19 severity [16, 17]. The CCI score was originally developed to predict the risk of mortality within 1 year of hospitalization [9]. It is a well-validated, simple, and easy-to-apply method for predicting prognosis and survival by estimating the risk of mortality from a comorbid disease. The application of CCI scoring in the context of the COVID-19 outbreak can be utilized for risk stratification [18].

Similar to many areas in China, inactivated vaccines manufactured by domestic pharmaceutical companies, mainly Sinovac and Sinopharm, were predominantly used in Shanghai and Suzhou. Breakthrough infection from the Omicron variant in fully vaccinated or booster recipients commonly occurred in our study, indicating possible vaccine escape. Nonetheless, vaccination remains a key strategy in controlling the COVID-19 pandemic. Although vaccines cannot completely prevent SARS-CoV-2 infection, they can significantly reduce clinical symptoms, hospitalizations, and deaths [4, 19]. Furthermore, accumulating evidence suggests that the duration of viral shedding of variants of concern is shorter in fully vaccinated recipients than in partially vaccinated or unvaccinated individuals [20, 21], which is consistent with our findings.

Nirmatrelvir/ritonavir and VV116, a remdesivir derivative, were included as new oral small-molecule antiviral drugs in our study; the former has an emergency use authorization from the US Food and Drug Administration [22], while the latter is currently undergoing clinical trials [15, 23, 24]. Ideally, the evaluation of protease inhibition for COVID-19 in high-risk patients (EPIC-HR) trial conducted by Hammond et al. showed that early treatment with nirmatrelvir/ritonavir could significantly reduce both the viral load and risk of COVID-19-related hospitalization or death [25]. Although trail enrollment was completed before the Omicron surge, an in vitro study that the Omicron variant is as susceptible to nirmatrelvir as the Delta variant [26]. Recent clinical studies further indicated that both the nirmatrelvir/ritonavir and VV116 can shorten the time of RT-PCR conversion in Chinese participants infected with the Omicron variant [15, 27]. Accordingly, our findings are consistent with those of previous studies, supporting the hypothesis that antiviral treatment reduces the risk of viral shedding in patients with the Omicron variant.

Taken together, clinicians and policymakers should focus on modifiable risk factors, such as providing booster vaccinations or effective antiviral therapy, which can shorten the duration of viral clearance and reduce the risk of transmission, rather than on fixed risk factors, such as age and underlying diseases. Assuming that the same patient had received booster vaccinations before becoming infected and was treated with effective antiviral agents after being infected (blue arrows, Fig. 4), the total number of points declined from 118 to 70. Therefore, there is a significant reduction in the probability of 14-day PCR conversion failure from 0.67 to 0.35 (blue lines, Fig. 4).

Additionally, these findings may contribute to developing and optimizing antiviral treatment strategies. Individuals with risk factors for delayed clearance of SARS-CoV-2 (i.e., older age, underlying disease, and incomplete vaccination) have an increased risk of progressing to severe or critical COVID-19. Currently, it is recommended that nirmatrelvir/ritonavir should be initiated within 5 days of onset and administered for 5 days. It may be necessary to extend the course of antiviral therapy in those with delayed virus clearance in order to reduce the possibility of viral rebound after withdrawal, and prevent disease progression. Further studies are required to confirm that extending the duration of treatment reduces the risk of viral rebound and disease progression.

Our study had some limitations. First, this was a retrospective study, and there may have been potential biases in patient selection. Second, symptom duration cannot be determined by reviewing medical records because symptoms were self-reported, potentially introducing bias. Third, the test for COVID-19 was based on RT-PCR and the corresponding Ct values in the present study. RT-PCR may be prone to false-negative and false-positive results, and a more reliable diagnosis can be achieved when combined with serologic tests [28]. Fourth, some participants’ estimated duration of viral RNA shedding may be longer than their actual duration, since viral RNA tests were not conducted daily. Fifth, Cox regression could not be used due some key variables violating the proportional hazards assumption. Therefore, we were unable to assess the hazard ratio for time to clearance of SARS-CoV-2. Finally, as the symptoms and hospitalization risk vary by subvariant [29, 30], and our study was restricted to the SARS-CoV-2 BA.2.2 subvariant [2], these findings may not be generalizable to other Omicron subvariants or SARS-CoV-2 variants.

## Conclusions

The proposed nomogram, based on four individual and clinical characteristics, accurately predicted 14-day PCR conversion failure in adults with non-severe COVID-19 during the Omicron surge. Hence, these results reinforce the guidance that elderly individuals, particularly those with increasing comorbidities, should be fully vaccinated against COVID-19 or receive a booster, as early as possible. Once infected, they should be treated with antiviral agents to shorten the duration of viral clearance and prevent disease progression.

## Data Availability

The datasets used and/or analyzed during the current study are available from the corresponding author on reasonable request.

## Abbreviations

AUC: Area under the ROC curve
CCI: Charlson comorbidity index
*CI*: Confidence interval
COVID-19: Coronavirus disease 2019
Ct: Cycle threshold
CT: Computed tomography
IQR: Interquartile range
*OR*: Odds ratio
ROC: Receiver operating characteristic
SARS-CoV-2: Severe acute respiratory syndrome coronavirus 2
VST: Viral shedding time
